# Self-Healing and Recyclable Polyurethane/Nanocellulose Elastomer Based on the Diels–Alder Reaction

**DOI:** 10.3390/polym16142029

**Published:** 2024-07-16

**Authors:** Tao Yang, Changhong Lin, Min Huang, Puyou Ying, Ping Zhang, Jianbo Wu, Tianle Wang, Alexander Kovalev, Nikolai Myshkin, Vladimir Levchenko

**Affiliations:** 1 International Joint Institute of Advanced Coating Technology, Taizhou University, Taizhou 318000, China; 2 Wenling Research Institute, Taizhou University, Taizhou 318000, China; 3 Zhejiang Provincial Key Laboratory for Cutting Tools, Taizhou University, Taizhou 318000, China; 4 School of Aeronautics, Zhejiang Institute of Communications, Hangzhou 311112, China; 5 Metal-Polymer Research Institute, National Academy of Sciences of Belarus, 246050 Gomel, Belarus

**Keywords:** polyurethane, epoxy resin, elastomer, Diels–Alder bond, recycling

## Abstract

With the background of the fossil fuel energy crisis, the development of self-healing and recyclable polymer materials has become a research hotspot. In this work, a kind of cross-linking agent with pendent furan groups was first prepared and then used to produce the Polyurethane elastomer based on Diels–Alder chemistry (EPU–DA). In addition, in order to further enhance the mechanical properties of the elastomer, cellulose nanofibers (CNFs) were added into the Polyurethane system to prepare a series of composites with various contents of CNF (wt% = 0.1~0.7). Herein, the FTIR and DSC were used to confirm structure and thermal reversible character. The tensile test also indicated that the addition of CNF increased the mechanical properties compared to the pure Polyurethane elastomer. Due to their reversible DA covalent bonds, the elastomer and composites were recycled under high-temperature conditions, which extends Polyurethane elastomers’ practical applications. Moreover, damaged coating can also be repaired, endowing this Polyurethane material with good potential for application in the field of metal protection.

## 1. Introduction

Since the emergence of polymeric materials, they have become one of the most important parts of our daily life. However, they have also caused some issues and risks for humankind, the environment, and ecological systems. So far, only a small fraction of polymer materials can be reused; most wasted polymer materials are discarded directly, which not only leads to the pollution and destruction of the environment and its ecology but also increases the cost of materials. As a consequence, the researchers developed several methods to repair damaged materials to lengthen their lifetime or to recycle polymer materials, like mechanical recycling, chemical, and biological catalysis, or using bio-based materials. Over the past thirty years, dynamic chemistry has provided a promising strategy for repairing and recycling polymer materials. In particular, thermoset materials with dynamic chemistry bonds can be reprocessed repeatedly, which has attracted wide attention from academia and industry and has become an important research subject for addressing the growing need for greener and more sustainable polymer materials [1,2]. Besides recycling ability, the dynamic chemistry bonds also endow polymer materials with self-healing capability after damage, which extends the service life, decreases the cost, and increases equipment security. Until now, the dynamic chemistry bonds for designing and developing self-healing and recyclable polymer materials can be divided into two categories; one is the dynamic covalent bonds (including oxime bonds [3], disulfide bonds [4], and Diels–Alder bonds [5]) and the other is the dynamic non-covalent bonds (such as coordinate bonds [6], hydrogen bonds [7], host–guest interactions [8], and electrostatic interactions [9]). These chemical bonds show their dynamic performance under certain stimuli (pH, light, temperature, magnetic fields, electricity, and mechanical force) [10], which make the polymer materials self-healing and recyclable. Moreover, in order to obtain self-healing polymer materials with comprehensive properties comparable to traditional polymer materials, the combination of multiple dynamic bonds is an important method [11,12,13]. Particularly, hydrogen bonds or multiple hydrogen bonds are often used as the physical cross-linking point combined with other dynamic bonds to play the role of enhancing and endowing self-healing ability [14,15,16].

Due to rich raw materials, adjustable structure, and phase separation structure, Polyurethane (PU) materials have excellent mechanical properties, chemical resistance, thermal stability, and anti-corrosion performance [17] and have gained significant attention in a wide range of fields, for instance, protection coating [18,19], medical devices [20], composites [21], damping materials [22], fire-retardant materials [23], etc. Therefore, the investigation of self-healing and recyclable Polyurethane materials has significance for practical applications. In order to balance self-healing efficiency and mechanical properties, scholars often use functional fillers (Graphene, Graphene oxide, carbon nanotubes). In polymer systems, these fillers cannot only enhance mechanical properties but also can respond to external stimuli and positively affect self-healing efficiency. Recently, the polymer-phase nanoenhancer based on microphase separation is another strategy to realize the high mechanical properties and high self-healing efficiency of self-healing materials. These include the “asymmetric dynamic hard segments” [24], “hard phase locking” [25,26], “local Phase-Lock Strategy” [27], and “dual hard-phase structures” [28]. High mechanical properties are achieved via the protecting/locking of multiple weak bonds in the hard phases. In addition, as a kind of renewable material that can replace petroleum-based materials, bio-based materials have been widely considered by researchers, such as lignin [29,30] and cellulose nanofibers (CNFs) [31]. Due to its high strength, stiffness, and biocompatibility, cellulose has been considered an ideal sustainable, functional nanomaterial to produce polymer composites. Moreover, cellulose has a number of functional groups which can accommodate various modifications. For example, TEMPO oxide cellulose nanofibers (TOCNFs) [32] and UPy–modified oxidized cellulose nanofibers (UTCNFs) not only strengthen the polymer matrix but also provide hydrogen bonds to obtain excellent self-healing, self-strengthening, and self-toughening abilities.

In this work, in consideration of its simple procedure and solid reaction without by-products, the Diels–Alder thermal reversible bond was used to produce the Polyurethane elastomer with proper mechanical properties and recyclability. On the one hand, the Epoxy segment with side furan units acted as the crosslinker to fix the Polyurethane segment, which was expected to obtain the high mechanical properties of elastomer. On the other hand, the CNF further enhanced the mechanical properties of Polyurethane. At the same time, we also studied the self-healing and protection of a Polyurethane elastomer coating.

## 2. Experimental Section

### 2.1. Materials

Isophorone diisocyanate (IPDI), Polytetrahydrofuran (PTMG, Mn (average) = 2000 g/mol), 2-Furfurylamine (FA), and dibutyltin dilaurate (DBTDL) were bought from Aladdin Reagent Co., Ltd. (Shanghai, China). The PTMG was dried at 100 °C for 2 h under vacuum before being used. Diglycidyl ether of bisphenol A (DGEBA, epoxide value: ~0.51 mol (100/g)) was supplied by Nantong Xingchen Synthetic Material Co., Ltd., (Nantong, China). The maleimide-functional compound N-hydroxyethyl maleimide (marked M-E) was prepared according to the reference [33]. The cellulose nanofibers (CNFs) were also provided by our lab. The anhydrous N,N-Dimethylformamide (DMF, AR) was purchased from Energy Chemical (Anhui Zesheng Technology Co., Ltd., Anqing, China). Other chemicals and solvents were used directly.

### 2.2. Preparation of Materials

#### 2.2.1. Synthesis of Furan-Pendent Epoxy

The Epoxy segment was prepared according to the report [34]. DGEBA (200 g) and anhydrous DMF (400 mL) were added into a three-neck flask equipped with a nitrogen inlet, followed by FA (68 g) which was added dropwise into the DGEBA solution. The reaction was conducted under magnetic stirring at 100 °C for 6 h. After reaction finished, the mixture was concentrated under reduced pressure and dried in a vacuum oven at 60 °C for 12 h to obtain a light-yellow viscous resin (named EP–FA).

#### 2.2.2. Synthesis of Maleimide End-Capped Polyurethane

The maleimide end-capped Polyurethane was synthesized via a two-step reaction using IPDI, PTMG2000, and M–E as the raw materials, according to the procedure previously reported by our group [35,36]. First, IPDI (26.68 g) and DMF (30 mL) were charged into a dried 500 mL round-bottomed flask. The PTMG (120 g)/DMF (120 mL) and an appropriate amount of DBTDL was added dropwise into the IPDI solution, and then the reaction was carried out at 80 °C for 3 h under magnetic stirring and nitrogen atmosphere to obtain the prepolymer. Then, M–E (16.92 g) dissolved in DMF (20 mL) was added dropwise into the above prepolymer. Once addition was finished, the mixture was stirred for another 8 h at 50 °C. After reaction was finished, the mixture was concentrated under reduced pressure and dried in a vacuum oven at 60 °C for 12 h to obtain a light-yellow viscous resin (marked as PU–M).

#### 2.2.3. Preparation of Diels–Alder Cross-Linked Elastomer and Composites

The CNF (0.01568 g) was first dispersed in DMF (30 mL) for 1 h by ultrasound to form a homogeneous dispersion. After that, the resulting EP–FA (3 g) and PU–M (12.68 g) were dissolved in prepared CNF dispersion, and the mole ratio of furan to maleimide was 1. Then, the mixture was poured into a PTFE mold and then placed in oven at 65 ℃ for 24 h to remove solvent and form the Diels–Alder cross-linked Polyurethane composites. The obtained sample was named EPU–DA–01CNF. The other materials with and without CNF were prepared according to the above method. The final samples were denoted as EPU–DA, EPU–DA–03CNF, EPU–DA–05CNF, and EPU–DA–07CNF, which represented CNF percentage concentrations of 0, 0.3, 0.5, and 0.7 wt%, respectively.

The formulation of all samples are listed in Table 1.

### 2.3. Characterization

The molecular weight and polydispersity (PDI) of prepared raw materials were determined by gel permeation chromatography (GPC) instrument (Waters, Waters e2695, Milford, MA, USA). The measurements were performed using THF with a flow rate of 1 mL/min at 35 ℃ and a series of narrow polystyrene standards for the calibration of the columns.

The FTIR spectrometer (Bruker Alpha II, Waltham, MA, USA) was used to verify the materials’ structure with scanning from 500 to 4000 cm^−1^.

The scanning electron microscope (SEM, Hitachi S-4800, Tokyo, Japan) was used to observe the morphology of CNF and polymer materials.

The thermogravimetric analyzer (TGA, Mettler-Toledo, Greifensee, Switzerland) was used to study the thermal stability behavior of Polyurethane materials. The measured temperature was carried out from room temperature to 800 °C at 20 °C min^−1^ under N_2_ condition.

The Differential scanning calorimetry (DSC, Netzsch SC200F3, Hanau, Germany) was performed to investigate dynamic thermal reversible behavior of samples under N_2_ atmosphere, from −30 °C to 200 °C, at a heating rate of 10 °C min^−1^.

The mechanical properties were evaluated using the universal tension machine (AI-7000-MU1, GOTECH testing Machines Inc., Dongguan, China) using a dumbbell-shaped sample (dimension: 35 mm × 2 mm × 0.5~1 mm) at room temperature at a rate of 2 mm min^−1^.

The programmable constant temperature and humidity chamber (KOMEG Technology Ind Co. Ltd, Dongguan, China) was used to analyze the resistance to moisture and heat aging, and the procedure was set at 60 °C and 90 relative humidity (RH) for 100 h.

## 3. Results and Discussion

### 3.1. Synthesis and Characterization of Elastomer and Composites

**Scheme 1:**
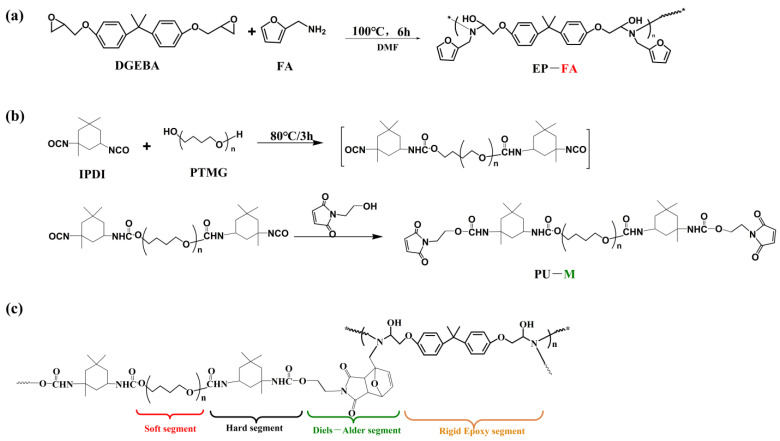
Schematic diagram of preparation procedure of (**a**) Epoxy with furan groups, (**b**) linear Polyurethane with maleimide groups, and (**c**) structure of polyurethane elastomer network.

In order to achieve the purpose of materials recycling, the Polyurethane elastomer was prepared based on the thermal reversible Diels–Alder mechanism. Scheme 1 depicts the synthesis process of the raw materials. First, the crosslinking agent was prepared using DGEBA and FA (Scheme 1a); the linear Polyurethane was produced via prepolymerization and the reaction of a maleimide-terminated compound and isophorone diisocyanate (Scheme 1b). Then, the molecular weight and polydispersity index (PDI) of EP–DA and PU–M were analyzed using gel permeation chromatography (GPC) in tetrahydrofuran (THF), and the results are shown in Table S1. The synthetic Polyurethane with maleimide groups (PU–M) and Epoxy resin with furan groups (EP–FA) were mixed according to the 1:1 molar ratio of furan-to-maleimide to prepare the cross-linked polymer elastomer (EPU–DA) (Scheme 1c). The Epoxy segment not only acted as the rigid phase together with the urethane group to strengthen the elastomer but also associated and disassociated through the Diels–Alder bonds.

**Figure 1:**
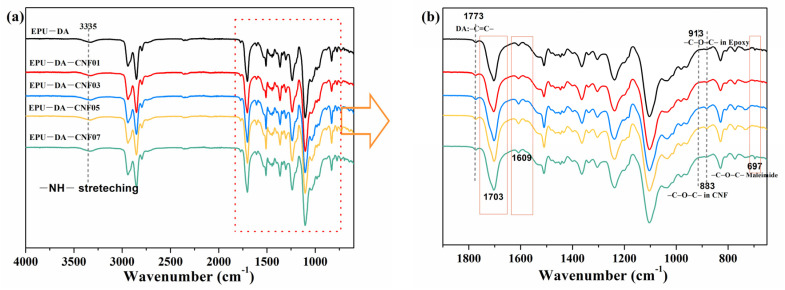
(**a**,**b**) FTIR spectra of pure EPU–DA and EPU–DA–CNF composites.

The FTIR spectra were used to identify the structure information and preparation process parameters of the elastomer and composites. The spectra of pre-polymer EP–FA, PU–M, and final materials EPU–DA and EPU–DA–CNF composites are shown in Figure S1 and Figure 1, respectively. It can be seen that the FTIR spectra of the EP–FA (Figure S1b) were characterized by the absence of a characteristic peak of the stretching vibrations of C–O–C of Epoxy groups at 913 cm^−1^. Moreover, the characteristic peak of the free –NCO group at 2270 cm^−1^ also appeared; the new characteristic peak of the furan ring at 732 cm^−1^, corresponding to the C=C bond of maleimide at 697 cm^−1^, was observed in the EP–FA and PU–M curves. These results display that the Epoxy group of DGEBA had completely ring-opening reactions [37], and the linear Polyurethane with maleimide groups was successfully synthesized.

In addition, from Figure 1a, the FTIR spectra of elastomer and composites show the characteristic peak of Polyurethane, including the stretching vibration of hydrogen-bonded –NH groups in the urethane of Polyurethane segment at 3335 cm^−1^, and the stretching vibration peaks of the C–H bond and CH_2_ from 3000 to2800 cm^−1^, respectively. The amplified absorbing peaks in the range of 1900–650 cm^−1^ are represented in Figure 1b. Compared with pure elastomer, the peak at 883 cm^−1^ was responsible for C–O–C glycosidic linkages between glucose units in cellulose [38]. Moreover, the intensity of this peak first increased and then decreased when increasing the CNF content. This phenomenon may be due to the CNF agglomerate in the EPU–DA–07CNF sample. The special peaks at 1609 cm^−1^ and 1705 cm^−1^ are associated with the amide II band (–NH), the stretching vibrations hydrogen-bonded –C=O groups in the urethane. More importantly, the character peak at 697 cm^−1^ (maleimide) and peak at 732 cm^−1^ (furan) disappeared; meanwhile, a characteristic shoulder peak at 1773 cm^−1^ in the elastomer and composites curves confirms the successful reaction between the furan and maleimide to form the DA bonds.

**Figure 2:**
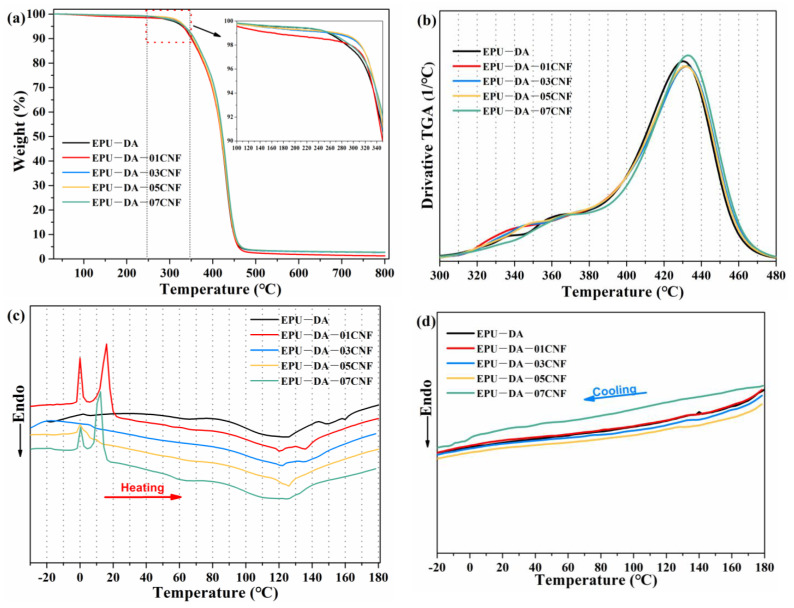
(**a**,**b**) TGA and DTG (**c**,**d**) DSC curves of pure EPU–DA and EPU-DA–CNF composites.

The thermal degradation behavior is of great importance during the development of thermal reversible cross-linked polymer materials as it provides guidance and evidence for the operating temperature for self-healing, reprocessing operations, and practical application. The thermal stability of EPU–DA and EPU–DA–CNF composites was studied by TGA from room temperature to 800 °C under an atmosphere of nitrogen. The thermogravimetric (TG) and derivative thermogravimetric (DTG) data of samples are shown in Figure 2a,b. For the elastomer and composites, there was an initial gentle decline in mass between the room and 240 °C, which mainly came from the solvent or heat-labile small-molecule evaporation. In general, the residual solvent or small molecules mainly act as the plasticizer in the polymer network, which decreases the comprehensive performance of materials, especially the mechanical properties. The next degradation stage, between 300 °C and 380 °C, is assigned to the degradation of a rigid segment of the Polyurethane system. In comparison with the EPU–DA, the degradation temperature of the composites with CNF increased during this stage. The large mass loss at about 410 °C is mainly assigned to the degradation of a soft segment of Polyurethane. The maximum degradation temperature of all samples has a negligible difference. As the CNF contains carbon-rich molecules, the mass residue of composites with different contents of CNF (0, 0.1, 0.3, 0.5, and 0.7 wt%) at 800 °C is significantly increased from 2.62 to 1.26, 2.65, 2.71, and 2.78 wt %, respectively.

For thermal-responsive materials, the association and dissociation temperatures are very important parameters for self-healing and for the reprocessed process. The DSC test was the most common means to investigate the thermal reversible behavior and the dissociation temperature of Diels–Alder bonds. The heating and cooling curves were displayed in the temperature range of −20 °C to 180 °C (Figure 2c,d). All samples showed the expected endothermal signals in the heating curves for rDA reaction between 80 and 140 °C. However, there are not any distinct exothermal peaks in the cooling curves because the furan and maleimide moieties do not have enough time for re-association during the cooling process [39]. The recovery of DA bonds needs more time in the solid state than solution state. Moreover, the EPU–DA exhibited characteristic endothermic peaks, including Tg of the hard segment (at about 50 °C) and Tm of the hard segment (160 °C). An additional endothermic peak at 125 °C would be associated with a less-ordered hard segment [40]. In the EPU–DA–01CNF, EPU–DA–05CNF, and EPU–DA–07CNF, the distinct cold crystallization and melting behavior can be found in the temperature ranges of 0–20 °C and 120–140 °C, which indicates these samples possess poor crystallization capacity and can hardly form crystalline structure at room temperature. However, the EPU–DA and EPU–DA–03CNF have no similar thermal transitions in their curves; this result may be due to the hard segment restricting the soft segment crystallization and formation of the crystalline structure in both their samples [41].

This cold crystallization phenomenon can be explained as follows: in a Polyurethane system, the formation of urethane and Diels–Alder linkages in a hard domain attempt to inhibit the soft segment crystallization behavior, and the degree of this reduction is affected by the relative content of hard and soft segments, and the size and morphology of hard domains. In the pure EPU–DA, only the hard segment constricts the crystallization of the soft segments. In composite samples, the CNFs play two roles: nucleating agent and inhibitor. When the content of CNFs is 0.1%, the role of the nucleating agent is dominant. When increasing the CNF content, the nucleating agent and inhibitor have a competitive relationship. In pure EPU–DA and EPU–DA–03CNF, entanglement between CNFs and the polymer chain reduces the activity of the soft segment and inhibit soft chain crystallization; hence, the CNF mainly play the inhibitor to crystallization. When the content of CNFs is increased to 0.7wt%, it is possible the aggregation effect is dominant in the EPU–DA–07CNF sample and caused the size of the hard domain to become smaller, so the cold crystallization was observed again.

### 3.2. Mechanical and Thermal Recyclable Properties of Elastomer and Composites

**Figure 3:**
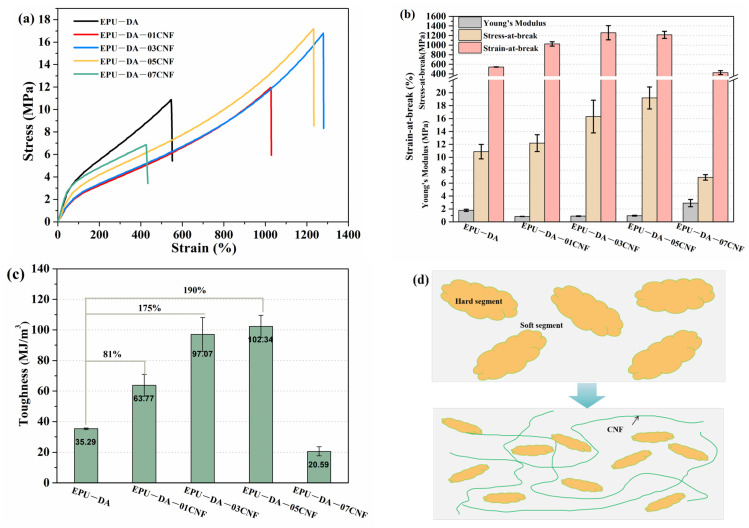
(**a**) The representative stress–strain curves, (**b**) summary of mechanical properties, and (**c**) toughness of pure elastomer and composites. (**d**) Schematic diagram of composites’ structure change.

The tensile stress–strain curves of all samples are shown in Figure 3a,b, and the results are summarized in Table S2. It can be seen that all samples show typical elastic tensile behavior. Owing to the enhancement effect of CNF, the composites have excellent mechanical strength and toughness compared to the pure EPU–DA sample. At a low CNF content, the addition of CNF is beneficial for improving the mechanical properties in the composites, that is, increasing the stress-at-break and strain-at-break from 10.88 MPa, 12.20 MPa, 16.31 MPa, 17.19 MPa, and 544%, 1024%, 1258%, 1215%, increasing the CNF content from 0.1 wt% to 0.5wt%. In particular, the toughness of EPU–DA–05CNF was higher by 190% than the EPU–DA elastomer (Figure 3c). However, the EPU–DA–07CNF had lower mechanical properties than pure EPU–DA, and the presence of excessive CNF will aggregate in the polymer matrix and then generate defects in the matrix, which cannot transfer the stress from the matrix to CNF. In addition, the elastomer modulus of CNF composites was decreased compared to the pure EPU–DA. This result may be related to the micro-scale structure morphology of CNF composites once the large aspect ratio nanocellulose was added into the EPU–DA system. The Diels–Alder covalent linkage between the Polyurethane chains and Epoxy resin chains was slightly decreased. Moreover, the size of the hard segment domain was reduced, and the level of phase mixing elevated [42] (Figure 3d); the DSC curves also indicated the cumulative enthalpy of fusion was decreased when increasing the CNFs.

**Figure 4:**
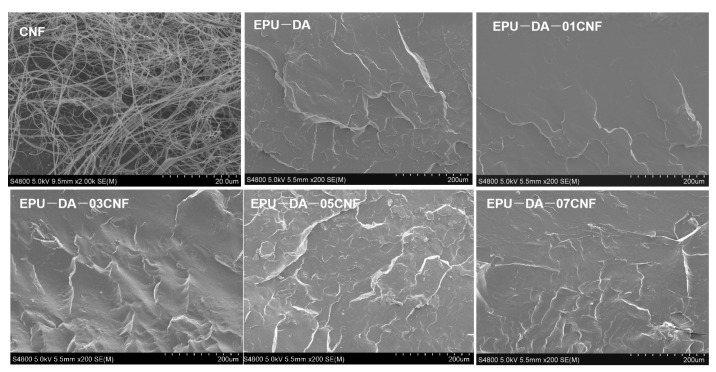
SEM image of CNF and Polyurethane materials.

The morphology of CNF and the cryogenically fracture surface of all samples are shown in Figure 4. Compared with other samples, the EPU–DA–05CNF show a rougher and corrugated surface, which shows this sample has high toughness.

**Scheme 2:**
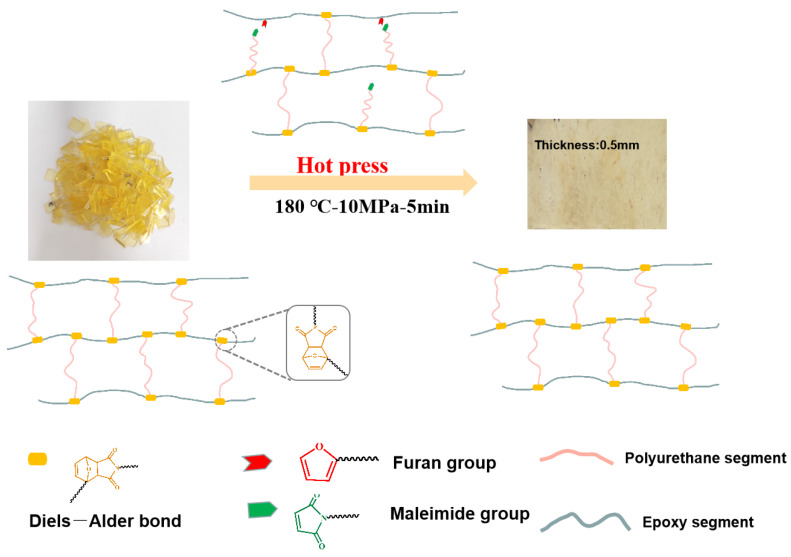
Schematic diagram of recycling of Polyurethane elastomer.

The thermal reversibility of Diels–Alder enables the Polyurethane elastomer to be reprocessed under heating conditions. To evaluate the recyclability of the Polyurethane elastomer, the samples were cut into small pieces, followed by placing them into a steel mold. The recycling process was conducted through hot pressing, in which the samples are firstly heated at 180 °C for 5 min with a pressure of 10 MPa, and then the mold is put into an oven at 65 °C for 24 h to ensure the recombination of the DA bonds. Scheme 2 presents the evolution of the cross-linked network.

**Figure 5:**
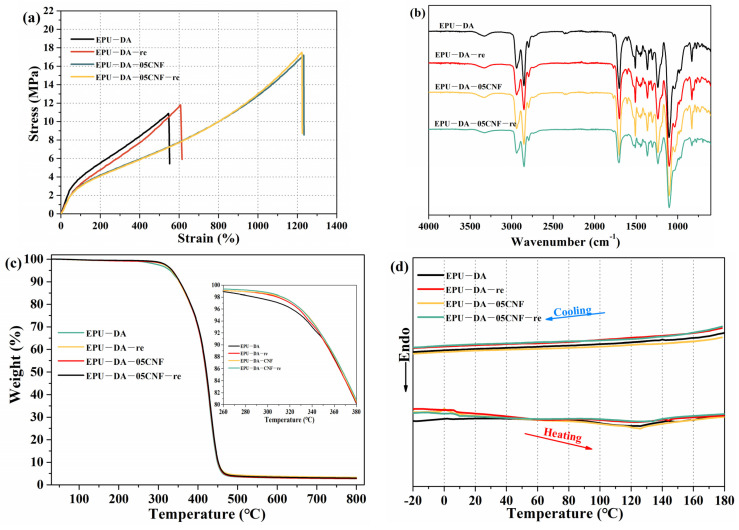
(**a**) Stress–strain curves, (**b**) FTIR, (**c**) TGA, and (d)DSC curves of recycled EPU–DA and EPU–DA–05CNF composites.

The EPU–DA and EPU–DA–05CNF were chosen to evaluate the DA cycle according to the above thermal treatment. In the following, the FTIR, TGA, DSC, and tensile tests were conducted to compare the structure and properties’ change before and after reprocessing (Figure 5a–d). After 24 h of healing at 65 °C, the recovered mechanical properties of the samples are given in Table S2 and Figure 5a. It was observed that the reprocessed samples can retain the mechanical properties, for instance, the tensile strength (10.36 MPa) and strain-at-break (554%) for the EPU–DA–re, and the tensile strength (20.14 MPa) and strain-at-break (1230%) for the EPU–DA–05CNF-re. In addition, compared with the original sample, the reprocessed samples have no new peaks in their FTIR spectra. The TGA curves of the recycled EPU–DA and EPU–DA–05CNF almost overlapped with those of the original sample. These results reveal that the elastomer and composites can be reprocessed and used like plastic materials without loss of performance.

### 3.3. Self-Healing and Protecting Experiments of Elastomer Coating

**Figure 6:**
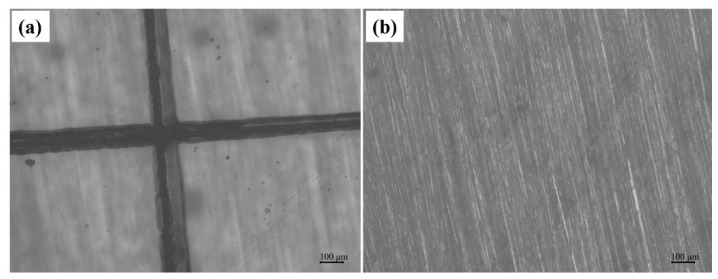
OM images of (**a**) a fresh crack (**b**) after being healed 10 min at 150 °C for EPU–DA coating.

The self-healing of protective coating for all kinds of substrates, such as protective coating for anti-corrosion coatings on metals, has greater advantages compared with the conventional protective coating, providing a more economical repair method by healing the scratched surface or the cracks repeatedly without loss of the coating materials, and extending the life of protective coatings [43,44]. Hence, we sprayed thermally self-healing elastomer coating on the 45# steel sheet. Then, the self-healing performance of the coating was evaluated according to the following procedure: cross scratches were made on the surface of the coating using a single-sided blade. Afterward, the damaged samples were heated at 150 °C for 10 min, then transferred to the oven at 65 °C for 24 h. An optical microscope was used to observe the micro-scratch evolution. It can be seen that the scratched area was found to be healed completely (Figure 6a,b). This healing mechanism can be explained: the heating treatment temperature (150 °C) is higher than the rDA bonds (120 °C), which then can accelerate the polymer chain movement and fill the crack. On the other side, the DA bonds re-associated under a low temperature (65 °C) and then recovered the support function of the coating.

In addition, temperature and moisture usually have adverse effects on the structure and properties of a coating, which in turn affects the protective effect. For this reason, it is necessary to evaluate the hydrothermal aging of the coating. Therefore, the specimens were placed into environment chambers to complete the accelerated hydrothermal aging process. Considering the thermal reversible property of the Diels–Alder bond, the temperature of the chamber during hydrothermal aging was kept at 60 °C, and the aging time was set to 100 h.

**Figure 7:**
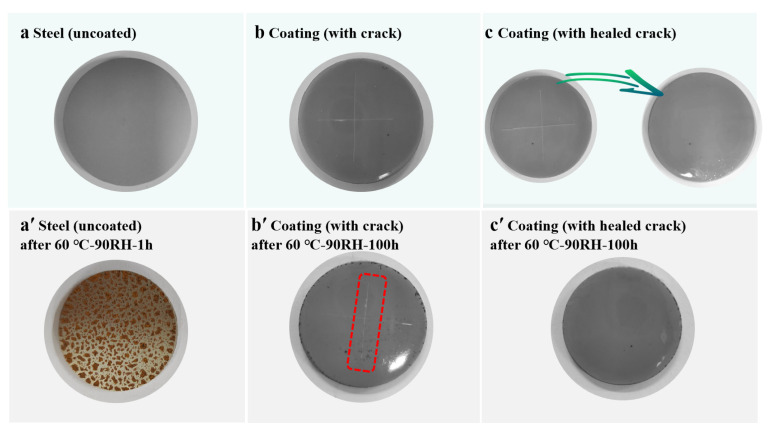
Digital photos of steel and steel with coating: (**a**,**a′**) steel (uncoated), (**b**,**b′**) coating (with crack), (**c**,**c′**) coating (with healed crack), before and after hydrothermal aging.

From Figure 7, it can be seen that some obvious corrosion spots quickly appeared on the surface of the steel sheet after 24 h, and some rust points also appeared on the surface of the coating (with crack) after 100 h, which indicated moisture had penetrated the surface of the steel, and the protective effect of the coating was destroyed. Compared with the above sample, the coating (with healed crack) still kept clean surface over the same period of time. This result shows that thermal self-healing performance plays an important role in protecting the substrate. Furthermore, the mechanical properties of pure elastomer and composites with hydrothermal aging were further investigated. As seen from the data in Figure S2 and Table S2, after 100 h continuous hydrothermal environment, the EPU–DA and EPU–DA–05CNF still maintained their structural integrity; the tensile strength of EPU–DA and EPU–DA–05CNF were 16.18% and 2.39% lower than that of the original sample. This result indicates the presence of CNF in the network can reduce water permeation and improve resistance to hydrothermal aging.

## 4. Conclusions

In this study, a self-healing and recyclable Polyurethane/CNF material with a Diels–Alder dynamic cross-linked network was developed. The structure was confirmed by FTIR and DSC. Due to both the rigid Epoxy segment and cellulose nanofibers acting as a polymer-enhancer and an inorganic-enhancer, the Polyurethane elastomer and composites had excellent mechanical properties, especially the 0.5 wt% CNF loading, which showed the highest tensile strength (17.19 MPa), 190% higher than pure elastomer. In addition, due to the intrinsic dynamic reversibility of the Diels–Alder bond, the elastomer and composites show excellent self-healing and recyclability. Furthermore, the Polyurethane coating also demonstrated excellent self-healing and anti-hydrothermal aging abilities. These results demonstrate its potential for application in the field of elastomer and elastomer coating.

## Figures and Tables

**Table 1:** Formulation of different samples.

Samples	EP–FA/g	PU–M/g	CNF/g
EPU–DA	3	12.68	0
EPU–DA–01CNF	3	12.68	0.01568
EPU–DA–03CNF	3	12.68	0.04704
EPU–DA–05CNF	3	12.68	0.0784
EPU–DA–07CNF	3	12.68	0.10976

## Data Availability

The original contributions presented in this study are included in the article/Supplementary Materials; further inquiries can be directed to the corresponding author/s.

## Supplementary Materials

The following supporting information can be downloaded at: https://www.mdpi.com/article/10.3390/polym16142029/s1, Table S1. Molecular weight and Polymer dispersity index (PDI) of EP-DA and PU-M. Table S2. Mechanical properties of samples. Figure S1 FTIR spectra of (a) DGEBA, (b) EP-FA and (c) PU-M. Figure S2 stress-strain curves of EPU-DA and EPU-DA-CNF before and after hydrothermal aging.
