# Supplementary material for: Self-Healing and Recyclable Polyurethane/Nanocellulose Elastomer Based on the Diels–Alder Reaction
Source: Polymers (Basel). 2024 Jul 16;16(14):2029. doi: 10.3390/polym16142029 (PMC11280686; doi:10.3390/polym16142029)
Supplement: Supplementary file 1 [file polymers-16-02029-s001.zip › polymers-3074354-supplementary.pdf]

# Self-healing and recyclable Polyurethane/Nanocellulose elastomer based on Diels-Alder reaction

Tao Yang[1,2,3]*, Changhong Lin[1,2,3], Min Huang[4], Puyou Ying[1,2,3], Ping Zhang[1,2,3], Jianbo Wu[1,2,3], Tianle Wang[1,2,3], Alexander Kovalev[1,2,3], Nikolai Myshkin[5], Vladimir Levchenko[1,2,3]**

1. International Joint Institute of Advanced Coating Technology, Taizhou University, Taizhou, Zhejiang 318000, China

2. Wenling Research Institute, Taizhou University, Taizhou, Zhejiang 318000, China

3. Zhejiang Provincial Key Laboratory for Cutting Tools, Taizhou University, Taizhou, Zhejiang 318000, China

4. School of Aeronautics, Zhejiang Institute of Communications, Hangzhou, Zhejiang 311112, China

5. Metal-Polymer Research Institute, National Academy of Sciences of Belarus, Gomel, 246050, Belarus

* Corresponding author. *E-mail address:* yangtaochd@163.com

** Corresponding author. *E-mail address:* vladlev@yahoo.com

Table S1. Molecular weight and Polymer dispersity index(PDI) of EP-DA and PU-M.

| Samples | Mn | Mw | PDI |
|---|---|---|---|
| EP-FA | 7665 | 14277 | 1.86 |
| PU-M | 6314 | 12318 | 1.95 |

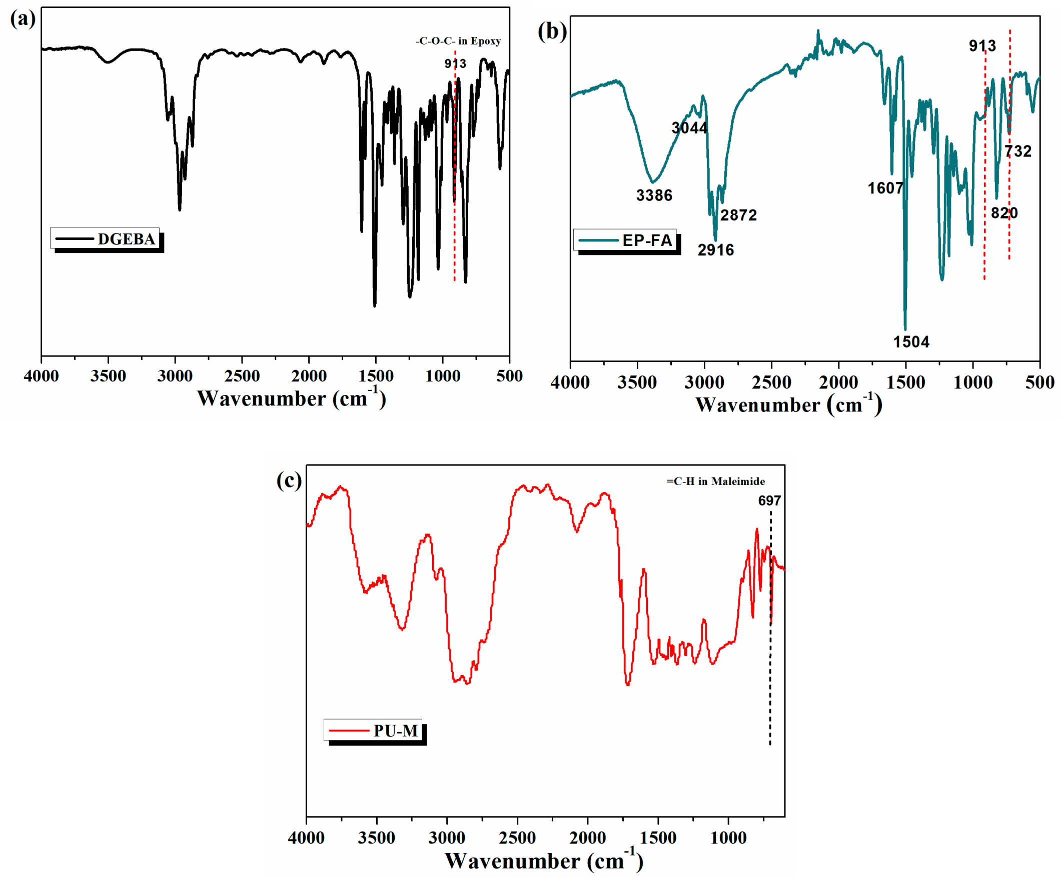


Figure S1 FTIR spectra of (a) DGEBA, (b) EP-FA and (c) PU-M

Table S2. Mechanical properties of samples.

| Samples | Stress-at-break [MPa] | Young's Modulus [MPa] | Strain-at-break [%] | Toughness [MJ/m$^3$] |
|---|---|---|---|---|
| EPU-DA | 10.88±0.34 | 1.79±0.18 | 544±4 | 35.29±0.52 |
| EPU-DA-01CNF | 12.20±1.31 | 0.84±0.02 | 1024±45 | 63.77±7.20 |
| EPU-DA-03CNF | 16.31±1.41 | 0.89±0.04 | 1258±100 | 97.07±11.07 |
| EPU-DA-05CNF | 17.19±1.23 | 0.96±0.07 | 1215±21 | 102.34±7.27 |
| EPU-DA-07CNF | 6.89±0.42 | 2.91±0.57 | 426±40 | 20.59±2.89 |
| EPU-DA-re | 10.36±1.71 | 1.56±0.12 | 554±75 | 33.09±8.34 |
| EPU-DA-05CNF-re | 20.14±1.69 | 1.26±0.09 | 1230±82 | 102.58±12.82 |
| EPU-DA-RH | 9.12±0.61 | 1.29±0.06 | 525±96 | 30.59±3.61 |
| EPU-DA-05CNF-RH | 16.78±2.28 | 0.85±0.08 | 1378±93 | 104.12±16.53 |

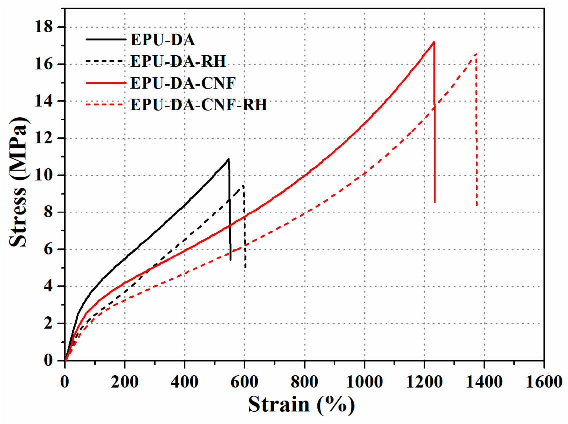


Figure S2 stress-strain curves of EPU-DA and EPU-DA-CNF before and after hydrothermal aging.
